# Invasive cervical cancers from women living in the United States or Botswana: differences in human papillomavirus type distribution

**DOI:** 10.1186/1750-9378-9-22

**Published:** 2014-07-08

**Authors:** Aaron Ermel, Doreen Ramogola-Masire, Nicola Zetola, Yan Tong, Brahim Qadadri, Marwan M Azar, Darron R Brown

**Affiliations:** 1 Department of Medicine, Indiana University School of Medicine, Indianapolis, IN, USA; 2 Botswana-University of Pennsylvania Partnership, Gaborone, Botswana; 3 Department of Biostatistics, Indiana University School of Medicine, Indianapolis, IN, USA; 4 Department of Microbiology and Immunology, Indiana University School of Medicine, Indianapolis, IN, USA; 5 Microbiology and Immunology, Division of Infectious Diseases, Indiana University School of Medicine, 635 Barnhill Drive, Room MS 224, Indianapolis, IN 46202, USA

**Keywords:** Human papillomavirus, Human immunodeficiency virus, DNA testing, Type distribution, Linear array

## Abstract

**Background:**

Cervical cancer is the primary cause of cancer-related deaths in women living in Botswana.

**Methods:**

Paraffin-embedded blocks of formalin-fixed invasive cervical cancer specimens were identified from women living in the U.S. (n = 50) or Botswana (n = 171) from which DNA was extracted. Thin-section PCR was performed on each sample for HPV types and HIV. Comparisons were made between HPV types and groups of types identified in cancers.

**Results:**

HPV DNA was identified in 92.0% of specimens from the U.S. containing amplifiable human DNA, and 79.5% of specimens from Botswana. HPV 16 was detected in 40 of 46 HPV-positive specimens (87.0%) from the U.S. vs. 58 of 136 (42.7%) from Botswana (p < 0.001). In contrast, non-HPV 16/18 types, all A9 species (HPV16, 31, 33, 35, 52, and 58), non-HPV 16 A9 (HPV 31, 33, 35, 52, and 58), HPV 18, all A7 types (18, 39, 45, 59, and 68) types were detected significantly more often in specimens from Botswana. The prevalence of non-HPV 18 A7 types did not differ significantly between the two groups. For specimens from Botswana, 31.6% contained PCR-amplifiable HIV sequences, compared to 3.9% in U.S. specimens. Stratifying the samples from Botswana by HIV status, HPV 31 was detected significantly more often in HIV-positive specimens. Other HPV types and groups of types were not significantly different between HIV-positive and HIV-negative specimens from Botswana.

**Conclusion:**

This study demonstrates that there may be important HPV type differences in invasive cervical cancers occurring in women living in the United States or Botswana. Factors in addition to HIV may be driving these differences.

## Introduction

Worldwide, cervical cancer is the second most common cancer in women and the most frequent cancer in women living in Sub-Sahara Africa, where the estimated incidence is 26.8 per 100,000 compared to 8.1 per 100,000 in the United States [1]. Two factors are believed responsible for this discrepancy: 1) cervical cancer screening is available in the U.S. but not in many Sub-Saharan African countries, and 2) possibly a higher prevalence of HIV infection in Sub-Saharan Africa. Infection with oncogenic, or “high risk” types of human papillomavirus (HR-HPV) is required for the development of cervical cancer, a malignancy that causes more than 400,000 deaths annually worldwide [2-5]. Previous studies indicate that HPV types 16 and 18 (HPV 16/18) together cause at least 70% of all cervical cancers; other HR-HPV types including 31, 33, 35, 39, 45, 51, 52, 56, 58, and 59 cause most of the remaining cervical cancers [4,6-8]. Current vaccines provide protection against infection and disease caused by HPV 16/18 but the cross protection against related types is not consistent. Second generation vaccines are currently in development. Determining HPV types in cervical cancers is critical in assessing needs for inclusion in second-generation vaccines. Therefore, a study was conducted to compare HPV type distribution in cervical cancer specimens from women living in either the United States or Botswana.

## Materials and methods

### Study specimens

Archived, paraffin-embedded blocks of formalin-fixed cervical cancer specimens were acquired from women residing in Indianapolis, Indiana (U.S.) or from throughout the country of Botswana. Cervical cancer specimens from the U.S. were collected during 2008 through 2011; those from Botswana between 2009 and 2010. Specimens from Botswana were re-embeded due to apparent dehydration of paraffin. Approval for this study was obtained through the Botswana Ministry of Health, Gaborone, Botwana and the Institutional Review Board at Indiana University School of Medicine, Indianapolis, IN. Sections (5 microns) were cut using a new blade for each block. The first and last sections were stained with hematoxylin and eosin for histopathologic classification.

Specimens with no epithelium were eliminated from the study. For specimens containing less than an estimated 5% of the total specimen, macrodissection was performed using a microscope to identify cancerous tissue, which was marked under the undersurface of the slide, then removed using a sterile scalpel.

### Thin-section PCR for HPV detection and typing

Four to six tissue sections were scraped from slides with a sterile blade. Tissue fragments were placed in 2 mL sterile tubes containing 1 mL octane. Tubes were rocked at room temperature for 10 minutes (min) and centrifuged at 19,000 × g for 10 min in a microcentrifuge. The octane supernatant was removed and fresh octane (500 uL) was added. Tubes were rocked for an additional 5 min to dissolve remaining paraffin. Samples were centrifuged at 19,000 × g for 5 min. The octane supernatant was removed and 1 mL of absolute ethanol was added to the pellet, and tubes were capped and inverted to mix, followed by centrifugation for 10 min at 19,000 × g. The supernatant was removed, 500 uL absolute ethanol was added to the pellet, then centrifugation was performed for 10 minutes at 19,000 × g. The ethanol was removed and pellets were dried in a Savant Speed Vacuum (Thermo Scientific, Waltham, MA) for 20 min.

Dried pellets were suspended in 300 uL Specimen Transport Medium (Qiagen, Hilden, Germany) and 10 μL proteinase K solution (20 μg/μL) was added (Qiagen). Samples were vortexed for 10 seconds and incubated at 55˚C overnight. DNA was extracted using the QIAamp MinElute Media Kit according to the manufacturer’s instructions (Qiagen). DNA concentration was determined by spectrophotometry (Beckman DU-64, Beckman Coulter, Brea, CA) and 200 ng was used for each PCR.

HPV detection and genotyping was performed using the Linear Array HPV Genotyping Test (LA-HPV) (Roche, Indianapolis, Indiana) as described previously [9-12]. The GH20/PC04 human β-globin target was co-amplified to determine sample adequacy. For specimens positive for β-globin but negative for HPV, DNA was extracted a second time from additional sections. If the specimen was again negative for β-globin, it was excluded in the analysis. If the specimen was positive for β-globin but negative for HPV a second time, it was considered HPV-negative.

### Thin-section PCR to detect HIV

Serum samples were not available, nor were medical records available for all subjects. Therefore, HIV was detected by thin-section PCR using DNA extracted from paraffin-embedded cervical cancer specimens. A 142 bp segment of the HIV-1 gag gene (nucleotides 1360-1507) was amplified using primers modified from An, SF et al [13]. Primer sequences were SK145-M (5′-GGG ACA TCA AGC CAT GCA AAT-3′) and SK431-M (5′-TGC TAT GTC AGT TCC CCT TGG TTC TCT-3′). A plasmid containing the gag and pol genes (pMDLg/pRRE) was used as a positive control (provided by Dr. Kenneth Cornetta, Indiana University School of Medicine, Indianapolis, IN). For PCR, 200 ng DNA template was mixed with 25 μL of Master Mix FastStart kit (Roche), 3 μM downstream and upstream primers, and distilled water for a total volume of 50 μL. Reactions conditions were: 1 cycle at 94°C for 9 min, then 40 cycles at 94°C for 30 seconds (denaturation), 55°C for 30 seconds (annealing), 72°C for 30 second (extension), followed by 1 cycle at 72°C for 7 min (final extension) in a Mastercycler ProS (Eppendorf, Hamburg, Germany).

PCR products, including controls were analyzed on 1.8% agarose gels. To confirm the amplification of desired products, two reactions from U.S. and Botswana cancers with an amplicon of expected size were extracted from gels using the QIAquick Gel Extraction Kit (Qiagen), then sequenced bi-directionally. Homology of the sequenced samples with known HIV gag sequences was determined using the Basic Local Sequencing Alignment Search Tool (BLAST) search (NCBI).

### Statistical analysis

Adequacy of extracted DNA based on β-globin-positivity among cancer specimens, and HPV-positivity among β-globin-positive samples was compared between U.S. and Botswana specimens. Type-specific HPV prevalence was then compared between U.S. and Botswana HPV-positive samples. HPV types were divided by their respective classification into Alpha 9 (A9 or HPV 16-related) and Alpha 7 (A7 or HPV 18-related). The A9 types included HPV 16, 31, 33, 35, 52, and 58, and the A7 types included HPV 18, 39, 45, 59, and 68. Prevalence of A9 and A7 types between U.S. and Botswana were compared. The difference in the distribution of cancers with multiple HPV types was compared between the sites of collection the U.S. and using a dichotomized approach (1 HPV type vs. more than 1 HPV type detected in a cancer). Additionally, HPV prevalence was compared between HIV-positive and HIV-negative specimens among Botswana samples. Comparisons were made using Fisher’s exact tests or Chi-square tests. Analyses were performed using SAS (Version 9.3, SAS Institute Inc., Cary, NC).

## Results

### Study specimens

Paraffin-embedded tissue blocks were obtained from 51 women from the U.S. (Table 1). All specimens from the U.S. met criteria for adequacy based on the amount of epithelium present as described above. From Botswana, paraffin-embedded tissue blocks were obtained from 214 women; 182 (85%) met adequacy criteria (Table 1). Only specimens with amplification of the human β-globin control were considered for analysis. Amplifiable DNA, as determined by positive amplification of the β-globin target, was extracted from 50 of 51 (98.0%) specimens from the U.S. and 171 of 182 (94.0%) specimens from Botswana (p = 0.472) (Table 1).

**Table 1:** Characteristics of cervical cancers from the U.S. and Botswana

**Study characteristics**	**Indiana, USA**	**Botswana**	**p-value**
Number of cancers with sufficient epithelium	51	182	--
β-globin positive (of cancer specimens)	50 (98.0%)	171 (94.0%)	p = 0.472^1^
HPV positive (of β-globin positive specimens)	46 (92%)	136 (79.5%)	p = 0.042^2^

^1^Fisher’s exact test, ^2^Chi-square test.

### Distribution of histopathologic types

All 50 cervical cancers from the U.S. were squamous cell carcinomas. From Botswana, 5 of the 171 (2.9%) cervical cancer specimens were adenocarcinomas and 166 of the 171 (97.1%) were squamous cell carcinomas.

### HPV types in cervical cancers

HPV (any type) was detected in 46 of the 50 (92.0%) β-globin-positive cancers from the U.S. and 136 of the 171 (79.5%) β-globin-positive cancers from Botswana (p = 0.042) (Table 1). HR-HPV types were detected in 46 of 46 (100%) HPV-positive specimens from the U.S. and 130 of 136 (95.6%) HPV-positive cancers from Botswana (p = 0.340) (Table 2). LR-HPV types were detected in 0 of 46 (0.0%) of HPV-positive cancers from the U.S. and 12 of 136 (8.8%) of HPV-positive cancers from Botswana (p = 0.039) (Table 2).

**Table 2:** Type-specific HPV detection in β-globin- and HPV-positive cervical cancers

**HPV Type**	**U.S., N = 46**	**Botswana, N = 136**	**p-value**
		**High-risk type distribution**	
HR-HPV	46 (100%)	130 (95.6%)	p = 0.340^1^
HPV 16	40 (87.0%)	58 (42.7%)	p < 0.001^2^
HPV 18	4 (8.7%)	32 (23.5%)	p = 0.029^2^
HPV26	0	5 (3.7%)	p = 0.332^1^
HPV31	1 (2.2%)	5 (3.7%)	p = 1.000^1^
HPV33	1 (2.2%)	7 (5.2%)	p = 0.682^1^
HPV35	0	8 (5.9%)	p = 0.205^1^
HPV39	0	6 (4.4%)	p = 0.340^1^
HPV45	3 (6.5%)	12 (8.8%)	p = 0.764^1^
HPV51	0	3 (2.2%)	p = 0.573^1^
HPV52	0	4 (2.9%)	p = 0.574^1^
HPV53	0	1 (0.7%)	p = 1.000^1^
HPV56	0	0	
HPV58	0	2 (1.5%)	p = 1.000^1^
HPV59	0	2 (1.5%)	p = 1.000^1^
HPV66	0	4 (2.9%)	p = 0.574^1^
HPV67	0	1 (0.7%)	p = 1.000^1^
HPV68	0	2 (1.5%)	p = 1.000^1^
HPV69	0	1 (0.7%)	p = 1.000^1^
HPV70	0	0	
HPV73	0	0	
HPV82	0	2 (1.5%)	p = 1.000^1^
HPVIS39	0	0	
		**Low-risk type distribution**	
LR-HPV	0	12 (8.8%)	p = 0.039^1^
HPV6	0	1 (0.7%)	p = 1.000^1^
HPV11	0	1 (0.7%)	p = 1.000^1^
HPV40	0	1 (0.7%)	p = 1.000^1^
HPV42	0	0	
HPV54	0	0	
HPV55	0	1 (0.7%)	p = 1.000^1^
HPV61	0	0	
HPV62	0	0	
HPV71	0	0	
HPV72	0	0	
HPV81	0	1 (0.7%)	p = 1.000^1^
HPV83	0	2 (1.5%)	p = 1.000^1^
HPV84	0	6 (4.4%)	p = 0.340^1^

^1^Fisher’s exact test, ^2^Chi-square test.

HPV 16 was the most frequently detected type in cancers from both countries (Table 2). HPV 16 was detected in 40 of 46 (87.0%) HPV-positive cancers from the U.S. and 58 of 136 (42.7%) HPV-positive cancers from Botswana (p < 0.001) (Table 2). In contrast, HPV 18 was detected in 4 of 46 (8.7%) HPV-positive cervical cancers from the U.S. and 32 of 136 (23.5%) HPV-positive cervical cancers from Botswana (p = 0.029) (Table 2).

Types other than HPV 16 and 18 were more often detected in cervical cancers from Botswana than from the U.S. (Table 3). Non-HPV 16/18 types were detected in 5 of 46 (10.9%) HPV-positive cancers from the U.S. and 65 of 136 (47.8%) HPV-positive cancers from Botswana (p < 0.001) (Table 3). A9 HPV types were detected in 41 of 46 (89.1%) HPV-positive cancers from the U.S. and 79 of 136 (58.1%) HPV-positive cancers from Botswana (p = 0.001) (Table 3). Non-16 A9 types (HPV 31, 33, 35, 52, and 58) were detected in 2 of 46 (4.4%) HPV-positive cancers from the U.S. and 26 of 136 (19.1%) HPV-positive cancers from Botswana (p = 0.016) (Table 3).

**Table 3:** Distribution of Alpha 9 and Alpha 7 types by location

	**U.S. (N = 46)**	**Botswana (N = 136)**	**p-value**
All Non HPV 16/18 types	5 (10.9%)	65 (47.8%)	p < 0.001
Non HPV 16/18 HR types	5 (10.9%)	56 (41.2%)	p = 0.002
All A9 Types^1^	41 (89.1%)	79 (58.1%)	p = 0.001
HPV 16	40 (87.0%)	58 (42.7%)	p < 0.001
Non-16 A9 Types	2 (4.4%)	26 (19.1%)	p = 0.016
All A7 Types^2^	7 (15.2%)	49 (36.0%)	p = 0.008
HPV 18	4 (8.7%)	32 (23.5%)	p = 0.029
Non-18 A7 Types	3 (6.5%)	18 (13.2%)	p = 0.218

All specimens positive for β-globin and HPV.

All analyses were performed using Chi-square tests.

^1^A9 types = HPV types 16, 31, 33, 35, 52, and 58.

^2^A7 types = HPV types 18, 39, 45, 59, and 68.

A7 HPV types were detected in 7 of 46 (15.2%) HPV-positive cervical cancers from the U.S. and 49 of 136 (36.0%) HPV-positive cancers from Botswana (p = 0.008) (Table 3). However, non-18 A7 types (39, 45, 59, and 68) were detected in 3 of 46 (6.5%) HPV-positive cancers from the U.S. and 18 of 136 (13.2%) HPV-positive cancers from Botswana, a non-significant difference (p = 0.218) (Table 3).

The distribution of HPV types in the 5 adenocarcinomas was HPV 18 in two cancers, HPV 16 in one cancer, HPV 26 in one cancer, and no HPV detected in one cancer.

### Representation of vaccine related HPV types

One or more HPV types represented in the two currently available vaccines (HPV type 6, 11, 16, or 18) were identified in 61.8% of cancers from Botswana and 93.5% of cancers from the U.S. A “second generation” vaccine is currently under development that may also protect against HPV types 31, 33, 45, 52, and 58 in addition to these four types. One or more of the nine types in the “second generation” vaccine were identified in 77.9% of cancers from Botswana and 100% of cancers from the U.S.

### HIV detection and HPV distribution in cervical cancers

The HIV sequences were amplified in 54 of 171 (31.6%) of cancer specimens with amplifiable DNA from Botswana and 2 of 50 (3.9%) of cancer specimens with amplifiable DNA from the U.S. (p < 0.001). A statistical analysis of HPV types by HIV status was performed on cervical cancers from Botswana, but not on cancers from the U.S. In the case of the two HIV positive cancers from the U.S., HPV 16 was detected in one and no HPV types were detected in the other.

The distribution of HPV types among HIV positive cancers and HIV negative cancers in Botswana is shown in Table 4. HPV 31 was detected in 4 of 44 (9.1%) HPV-positive cervical cancer specimens from HIV-positive women, and 1 of 92 (1.1%) HPV-positive cervical cancer specimens from HIV-negative women (P = 0.038). HR-HPV types were detected in 41 of 44 (93.2%) HPV-positive cervical cancer specimens from HIV-positive women, and 89 of 92 (96.7%) HPV-positive cervical cancer specimens from HIV-negative women (p = 0.388). The distributions of LR-HPV, HPV 16 and 18, non-HPV 16/18, non-HPV 16/18 HR-HPV types, A7 or A9 groups, non-HPV 16 A9 types, and non-HPV 18 A7 types did not differ significantly between cervical cancers from HIV-positive and HIV-negative women (Table 5). No cervical cancers from the U.S. and 6 cervical cancers from Botswana contained only a LR-HPV type. Three of these cancers were from women whose cancers tested HIV-positive; HPV 84 was detected in 2; HPV 40 in 1 specimen. Three were from women whose cancers tested HIV-negative; all 3 contained HPV 84.

**Table 4:** HPV types in β-globin- and HPV-positive cervical cancers from Botswana by HIV status

**HPV Type**	**HIV-pos, N = 44**	**HIV-neg, N = 92**	**p-value**
		**High-risk type distribution**	
HPV 16	20 (45.5%)	38 (41.3%)	p < 0.647^2^
HPV 18	8 (18.2%)	24 (26.1%)	p = 0.309^2^
HPV26	0	5 (5.4%)	p = 0.174^1^
HPV31	4 (9.1%)	1 (1.1%)	p = 0.038^1^
HPV33	2 (4.6%)	5 (5.5%)	p = 1.000^1^
HPV35	4 (9.1%)	4 (4.4%)	p = 0.273^1^
HPV39	3 (6.8%)	3 (3.3%)	p = 0.388^1^
HPV45	2 (4.6%)	10 (10.9%)	p = 0.336^1^
HPV51	0	3 (3.3%)	p = 0.551^1^
HPV52	0	4 (4.4%)	p = 0.304^1^
HPV53	0	1 (1.1%)	p = 1.000^1^
HPV56	0	0	
HPV58	0	2 (2.2%)	p = 1.000^1^
HPV59	0	2 (2.2%)	p = 1.000^1^
HPV66	2 (4.6%)	2 (2.2%)	p = 0.595^1^
HPV67	0	1 (1.1%)	p = 1.000^1^
HPV68	0	2 (2.2%)	p = 1.000^1^
HPV69	0	1 (1.1%)	p = 1.000^1^
HPV70	0	0	
HPV73	0	0	
HPV82	0	2 (2.2%)	p = 1.000^1^
HPVIS39	0	0	
		**Low-risk type distribution**	
HPV6	0	1 (1.1%)	p = 1.000^1^
HPV11	0	1 (1.1%)	p = 1.000^1^
HPV40	1 (2.3%)	0	p = 0.324^1^
HPV42	0	0	
HPV54	0	0	
HPV55	0	1 (1.1%)	p = 1.000^1^
HPV61	0	0	
HPV62	0	0	
HPV71	0	0	
HPV72	0	0	
HPV81	1 (2.3%)	0	p = 0.324^1^
HPV83	0	2 (2.2%)	p = 1.000^1^
HPV84	2 (4.6%)	4 (4.4%)	p = 1.000^1^

^1^Fisher’s exact test, ^2^Chi-square test.

**Table 5:** HPV type distribution by HIV status in cervical cancer specimens from women living in Botswana by HR-HPV, LR-HPV and clade

	**HIV Positive, N = 44**	**HIV Negative, N = 92**	**p-value**
Any HPV type	44 (100%)	92 (100%)	---------
HR-HPV^1^	41 (93.2%)	89 (96.7%)	p = 0.388^5^
LR-HPV^2^	4 (9.1%)	8 (8.7%)	p = 1.000^5^
All non-16/18 HPV types	20 (45.5%)	45 (48.9%)	p = 0.706^6^
All non-16/18 HR- HPV types	17 (38.6%)	39 (42.4%)	p = 0.677^6^
All A9 types^3^	29 (65.9%)	50 (54.4%)	p = 0.201^6^
HPV 16 positive	20 (45.5%)	38 (41.3%)	p = 0.647^6^
Non-16 A9 types	10 (22.7%)	16 (17.4%)	p = 0.459^6^
All A7 types^4^	13 (29.6%)	36 (39.1%)	p = 0.276^6^
HPV 18 positive	8 (18.2%)	24 (26.1%)	p = 0.309^6^
Non-18 A7 types	5 (11.4%)	13 (14.1%)	p = 0.656^6^

All specimens positive for β-globin and HPV.

^1^HR-HPV = High-risk HPV types,

^2^LR-HPV = Low-risk HPV types,

^3^A9 types = HPV types 16, 31, 33, 35, 52, and 58;

^4^A7 types = HPV types 18, 39, 45, 59, and 68;

^5^Fisher’s exact test;

^6^Chi-square test.

### Distribution of multiple HPV types in cervical cancers

All HPV-positive cancers were compared between countries for detection of multiple HPV types. A single HPV type was detected in 43 of 46 (93.5%) HPV-positive U.S. cervical cancers and 113 of 136 (83.1%) HPV-positive cervical cancers from Botswana (p < 0.082). The distribution of HPV types among cancers containing single vs. multiple HPV types by country is shown in Table 6. HPV 16 was identified as a single isolate in 92.5% of cancers from the U.S. and 74.1% of cancers from Botswana (p = 0.021). No other HPV types differed significantly in terms of single or multiple isolates between cancers from the two countries.

**Table 6:** Detection of HPV as single or multiples types in β-globin- and HPV-positive cervical cancers

	**Botswana**		**U.S.**	
	**Single type**	**Multiple types**	**Single type**	**Multiple types**
**High-risk HPV types**				
HPV 16*	43 (74.1%)	15 (25.9%)	37 (92.5%)	3 (7.5%)
HPV 18	23 (71.9%)	9 (28.1%)	3 (75.0%)	1 (25.0%)
HPV26	3 (60.0%)	2 (40.0%)	0 (0.0%)	0 (0.0%)
HPV31	5 (100.0%)	0 (0.0%)	1 (100.0%)	0 (0.0%)
HPV33	5 (71.4%)	2 (28.6%)	0 (0.0%)	1 (100.0%)
HPV35	6 (75.0%)	2 (25.0%)	0 (0.0%)	0 (0.0%)
HPV39	4 (66.7%)	2 (33.3%)	0 (0.0%)	0 (0.0%)
HPV45	8 (66.7%)	4 (33.3%)	2 (66.7%)	1 (33.3%)
HPV51	1 (33.3%)	2 (66.7%)	0 (0.0%)	0 (0.0%)
HPV52	2 (50.0%)	2 (50.0%)	0 (0.0%)	0 (0.0%)
HPV53	0 (0.0%)	1 (100.0%)	0 (0.0%)	0 (0.0%)
HPV56	0 (0.0%)	0 (0.0%)	0 (0.0%)	0 (0.0%)
HPV58	1 (50.0%)	1 (50.0%)	0 (0.0%)	0 (0.0%)
HPV59	1 (50.0%)	1 (50.0%)	0 (0.0%)	0 (0.0%)
HPV66	3 (75.0%)	1 (25.0%)	0 (0.0%)	0 (0.0%)
HPV67	0 (0.0%)	1 (100.0%)	0 (0.0%)	0 (0.0%)
HPV68	1 (50.0%)	1 (50.0%)	0 (0.0%)	0 (0.0%)
HPV69	0 (0.0%)	1 (100.0%)	0 (0.0%)	0 (0.0%)
HPV70	0 (0.0%)	0 (0.0%)	0 (0.0%)	0 (0.0%)
HPV73	0 (0.0%)	0 (0.0%)	0 (0.0%)	0 (0.0%)
HPV82	1 (50.0%)	1 (50.0%)	0 (0.0%)	0 (0.0%)
HPVIS39	0 (0.0%)	0 (0.0%)	0 (0.0%)	0 (0.0%)
**Low-risk HPV types**				
HPV6	0 (0.0%)	1 (100.0%)	0 (0.0%)	0 (0.0%)
HPV11	0 (0.0%)	1 (100.0%)	0 (0.0%)	0 (0.0%)
HPV40	1 (100.0%)	0 (0.0%)	0 (0.0%)	0 (0.0%)
HPV42	0 (0.0%)	0 (0.0%)	0 (0.0%)	0 (0.0%)
HPV54	0 (0.0%)	0 (0.0%)	0 (0.0%)	0 (0.0%)
HPV55	0 (0.0%)	1 (100.0%)	0 (0.0%)	0 (0.0%)
HPV61	0 (0.0%)	0 (0.0%)	0 (0.0%)	0 (0.0%)
HPV62	0 (0.0%)	0 (0.0%)	0 (0.0%)	0 (0.0%)
HPV71	0 (0.0%)	0 (0.0%)	0 (0.0%)	0 (0.0%)
HPV72	0 (0.0%)	0 (0.0%)	0 (0.0%)	0 (0.0%)
HPV81	0 (0.0%)	1 (100.0%)	0 (0.0%)	0 (0.0%)
HPV83	0 (0.0%)	2 (100.0%)	0 (0.0%)	0 (0.0%)
HPV84	5 (83.3%)	1 (16.7%)	0 (0.0%)	0 (0.0%)

*The difference between multiple type detection in the two countries was statistically significant for HPV 16 only (p = 0.021.). All other comparisons were not statistically significant.

For the 3 U.S. specimens that contained more than one type, all contained a total of 2 types. For the 23 specimens from Botswana that contained more than one type, 19 contained 2 types, 2 contained 3 types, 1 contained 4 types, and 1 contained 7 types. The HPV-positive cervical cancer specimens from Botswana were then stratified by HIV status. More than 1 HPV type was detected in 5 of 44 (11.4%) of cervical cancers testing HIV-positive and 18 of 92 (19.6%) of cervical cancers from lesions testing HIV-negative; a non-significant difference (p = 0.233).

## Discussion

Cervical cancer is the most frequent cancer in women living in Sub-Sahara Africa. High quality data on HPV types in invasive cervical cancers from African women is lacking, because most studies utilized exfoliated cells from cervical swabs for type determination rather than actual cervical cancer tissue. Though useful in understanding the overall epidemiology of HPV in Africa, such studies do not provide an accurate representation of HPV types in cervical cancers. In the current study, a direct approach (thin-section PCR) was used to isolate DNA from formalin-fixed invasive cancer specimens.

HPV 16 was the most frequently detected type in invasive cervical cancers from women living in the U.S. (87% of all cancers). HPV 16 was also the most frequently detected HPV type in cancers from women living in Botswana, but was significantly less often detected in this group (42.7% of all cancers) compared to the U.S. HPV 18 was less frequently detected in cancers from women living in the U.S. (8.7% of all cancers), compared to 23.5% in cancers from women living in Botswana. The combined frequency of HPV 16/18 detection in cancers from the U.S. was 93.5%, compared to a combined frequency of HPV 16/18 of 61.8% in cancers from Botswana. Overall, non-HPV 16 A9 family members were significantly more frequently detected in cancers from women living in Botswana, but the prevalence of non-HPV 18 A7 family members did not differ significantly between the U.S. and Botswana.

HIV is a factor that likely contributes to differences in HPV types in cancers [14-16]. Stratification of cancers from Botswana (and the HPV types detected) by HIV status showed no significant differences except for HPV 31, which was found more frequently in HIV-positive than HIV-negative women from Botswana. Thus, it cannot be stated that HIV was the *only* factor driving differences in HPV types in cervical cancers from women living in Botswana. However, factors related to HIV infection (i.e., duration of infection and CD4 counts) were not analyzed in our study and may influence the HPV type distribution. Other factors to consider in future studies include geographical variation, nutritional factors and co-infections with other pathogens.

What have other studies shown regarding HPV types in cervical cancers in African women? One study, utilizing cervical swabs from HIV-positive women living in Botswana with cervical intraepithelial neoplasia (CIN) grades 2 or 3 showed that a range of HPV types could be detected by PCR. Overall, 56% of these women were infected with multiple HR-HPV types. The most common HPV types found in women with CIN2 and CIN3 were HPV 16 and 18 accounting for 51% of their subjects [17]. In another study, De Vuyst *et al*., examined invasive cervical carcinoma specimens from women living in Kenya or South Africa, and found that the combined percentage of HPV 16 plus HPV 18 was approximately 68%, a percentage close to that presented in this report for women living in Botswana [18]. No significant difference was found between HIV-positive or HIV-negative women for HPV 16/18 positivity. Denny et al. examined HPV types in invasive cervical cancers from women living in two western African countries (Ghana and Nigeria) and one south African country (South Africa) [19]. This study utilized formalin-fixed, paraffin-embedded sections of cervical cancers to perform thin section PCR, using the SPF_10_ DEIA-LiPA_25_ system. They found that HPV 16 was detected in 50.7% of cancers and HPV 18 in 19.2%. Similar to our study, multiple HPV types were detected in only 11.1% of cancers. HIV status was not known for most of the women in this study.

In the current study, there were cervical cancers in which more than a single HPV type was detected. Cervical cancer is a clonal process, and no studies have demonstrated more than one HPV type in a single cell. However, cervical lesions have been demonstrated that contain separate dysplastic foci, each containing different HR-HPV types [20]. One explanation is that HIV-positive women have multiple foci of disease infected with different HR-HPV types. However in our study, there was no significant difference in the distribution of multiple HPV types being detected between HIV infected and HIV uninfected individuals.

There are several limitations to this study. First, only one U.S. site and one African site were included, so these results cannot be generalized. Second, the study used paraffin-embedded specimens, and as a result, the sensitivity for HPV detection may be less than that if fresh cancer tissues had been available. It is possible that the techniques used to detect and type HPV have different performance capabilities for certain types, but the Roche Linear Array has become the standard typing method in epidemiologic studies. Certain conditions of fixation affect the recovery of DNA from paraffin-embedded tissues [21,22]. Because the current study used archived specimens, fixation conditions were not controlled and may have contributed to differences in HPV detection. Third, only ~80% of the Botswana samples with adequate epithelium histologically were eventually analyzed. Given the size of the study, a few cases could shift the results. Lastly, the HIV serostatus of the women was not known. An indirect method (amplification of HIV DNA from paraffin-embedded tissues) was used to determine HIV status, and the sensitivity of this method is unknown. The prevalence of HIV in women in the current study, as determined by this method, was 29.9%, a number comparable to the national prevalence of 23.4% in adults living in Botswana [23]. However, some studies have noted a prevalence of HIV of nearly 60% when looking at women with invasive cervical cancer in this clinic (unpublished data, but personally communicated by Doreen Ramogola-Masire). Since HIV infected women may progress more frequently to cancer, there may have been an over-representation of HIV-positive women compared to the general population of Botswana, but testing for HIV proviral DNA may be nit be sensitive enough to detect all HIV positive samples. This study also lacks demographic and behavioral data (sexual practices, partner number, etc.), which makes it difficult to apply these results to the general population.

In summary, amplifiable DNA was extracted from archived invasive cervical cancers from women living in the U.S. or Botswana. The contribution of HPV types 16 and 18 was significantly higher in cancers from the U.S. compared to cancers from Botswana. Conversely, non-HPV 16/18 oncogenic types were more frequently detected in cervical cancers from Botswana. Multiple HPV types were infrequently identified in cancers from either country. HIV status had a modest effect on HPV type distribution in cervical cancers from women living in Botswana. In larger future studies, a more pronounced effect of HIV on HPV types detected in invasive cervical cancers may be evident. Because current vaccines against HPV do not provide reliable or lasting protection against oncogenic types other than HPV 16 and HPV 18, such studies are important to conduct in other African countries.

## Competing interest

Darron R. Brown, MD received lecture fees, advisory board fees, and intellectual property fees from Merck and Co.

## Authors’ contributions

AE- manuscript preparation, laboratory methods, data analysis and submission. DRM- study design, procurement of samples, manuscript preparation. NZ- data analysis and manuscript preparation. YT- data and statistical analysis and manuscript preparation. BQ- laboratory methods, testing of samples, and manuscript preparation. MA- testing of samples, data analysis and manuscript preparation. DB- principal investigator, study design, data analysis, and manuscript preparation. All authors read and approved the final manuscript.
